# Digital psychosocial interventions for individuals with spinal cord injury: a scoping review

**DOI:** 10.3389/fpsyt.2024.1289138

**Published:** 2024-01-22

**Authors:** Alice Armstrong, Katja Oetinger, Katja Weimer, Klaus Hönig

**Affiliations:** Department of Psychosomatic Medicine and Psychotherapy, Ulm University Medical Centre, Ulm, Germany

**Keywords:** spinal cord injury, paraplegia, digital intervention, digital health, psychosocial intervention, medical apps, mhealth

## Abstract

**Objective:**

To provide an overview of the digital mental health care landscape for individuals with spinal cord injury (SCI).

**Methods:**

PubMed, PsycInfo, and PSYNDEX were searched for articles meeting the following criteria: (1) article written in English or German; (2) digital psychosocial intervention; (3) SCI only; (4) treatment of individuals with SCI and not their relatives or caregivers. Records were screened by title and abstract and records meeting the inclusion criteria were obtained for full text screening. The references of identified articles were screened to find further relevant articles. The literature search was updated before submission. Risk of Bias was assessed by using the Cochrane risk-of-bias tool for randomized trials (RoB 2) and a narrative synthesis was conducted.

**Results:**

Ten randomized-controlled trials (RCT) and ten non-randomized-controlled trials were identified and compared in this review, evaluating twelve internet- and mobile-based interventions, five smartphone apps, and three virtual reality applications. The interventions were primarily used as stand-alone aftercare programs. While some were not based on any theory, cognitive behavioral therapy mostly served as the theoretical basis for the online interventions. The extent of human support also varied greatly between the studies. The number of intervention modules ranged between 2 and 72. There were also major differences in outcome variables and effects. A meta-analytical evaluation of the data was not conducted due to heterogeneity of studies.

**Conclusion:**

Digital applications to promote the psychosocial health of individuals with SCI are an emerging field of research with many treatment approaches still to come. First high quality RCT studies report promising results. Unfortunately, not all studies are of high quality or the interventions have been insufficiently adapted to the needs of people with SCI. Therefore, more research is needed to further develop applications, and to generalize and test the effects found in the long term.

## 1 Introduction

Spinal cord injury (SCI) poses great psychosocial challenges for the person affected and requires a long-lasting adaptation process which is reflected, among other things, in an above-average mental morbidity (1–5). Social support in the sense of the bio-psycho-social model plays a major role in coping processes (6). Perceived social support from family, friends and peers influences the level of acceptance of the disability and has positive effects on pain, subjective well-being, and mental health (7, 8). However, compared to the general population, people with SCI are more likely to feel lonely (9), are more frequently single (10) and have higher divorce rates (11) after the injury.

Due to the above-average mental morbidity and the lack of social support, professional support is particularly relevant for people with SCI. However, the professional support provided so far has not been sufficient. This statement is being supported by the study of Fann et al. (12): in persons with SCI (*N* = 947), 23% suffered from probable major depression (PHQ-9 score ≥ 10). However, in depressed persons with SCI only 11% received antidepressant treatment according to guidelines and 6% had been in guideline-level psychotherapy treatment in the past three months. This is fewer than in the general population (12). This could be due to the severity of the illness: various challenges exist, for example, limited mobility because of a wheelchair, lack of psychosocial support or the need for an assistant (8, 10). Furthermore, barrier-free counselling rooms and accessible travel options are mandatory for individuals with SCI to receive adequate psychotherapeutic treatment (13).

As conventional treatment options are not suitable for all people with SCI due to the factors mentioned above (12), innovative digital applications could be used as an extension to the current psychosocial care (14). Digital interventions can overcome both structural barriers (lack of healthcare services, long waiting lists, high costs) and physical barriers (steps, too narrow door frames, inappropriate toilet facilities) to adequate face-to-face treatments (8, 14). Therefore, digital interventions have the potential to lower the psychosocial care gap in persons with SCI.

The term digital interventions covers a wide range of applications, which can differ from one another in various aspects. According to Paganini et al. (15), digital interventions can be categorized according to four characteristics: indication and application areas, theory basis, human support and technology. *Indication & application areas* refers to whether the intervention is used as a preventive, acute or aftercare measure or for relapse prevention. Furthermore, in contrast to a “stand-alone” intervention, where only the self-help program is used by the patient, the option of “blended care” offers a combination of digital interventions with traditional psychotherapy in person. *Theory basis* refers to whether the content of the digital intervention is based on an established psychotherapeutic procedure. Because of their standardization and modularization, therapies such as cognitive behavioral therapy (CBT) or acceptance and commitment therapy (ACT) are suitable as digital intervention. The category *human support* differs between “unguided” and “guided” interventions. In the latter, for example, participants receive feedback and motivation, usually from professional coaches. The *technology* aspect shows the various ways in which digital interventions can be implemented (e. g. audio files, video files, chat rooms, text messages, etc.) (15).

This article focuses on three types of psychosocial digital interventions: internet and mobile-based interventions (IMIs), mobile apps for smartphones and virtual reality applications.

Firstly, IMIs are primarily self-help interventions based on instructive online programs that are made available via a website and are used by people on health-related topics (16). IMIs intend to have a desirable effect on the mental health, e. g. depression, and quality of life of users (17, 18), and hence must be evaluated thoroughly. The effectiveness of IMIs has been demonstrated through high effect sizes for the improvement of various mental disorders in the general population (15). For example, in a meta-analysis for IMIs on depression and anxiety, an average effect size of *g* = 0.88 was found (19). Individuals with SCI are also affected by such mental disorders, but because of the barriers mentioned above, IMIs are especially attractive for this group. However, as the market for digital applications is wide, with strong qualitative differences, research needs to evaluate the content of such IMIs (15).

Secondly, mobile health (mhealth) apps form another category of digital interventions in which therapeutic content is being conveyed via the smartphone (20). In general, studies on apps that addressed anxiety or depressive symptoms were of medium to high quality and generally had small to medium effect sizes (20). This range of effect sizes was also found for apps that examined the effects on stress and quality of life (20). As people with SCI have increased scores in depression, anxiety and stress (21), it is interesting to examine the apps available for this target group.

Lastly, in virtual reality applications the user is immersed in a computer-generated, three-dimensional world (22). Initial results show that these applications can be successful in relation to various psychopathologies such as phobias, post-traumatic stress disorders and psychological stress (23). Favorable effects were also found in depression, anxiety, and pain experience (23, 24). The fact that desirable effects were found in various psychological conditions suggests that individuals with SCI could also experience positive effects from VR-interventions. For example, relief of neuropathic pain using VR-applications such as virtual walking, or virtual illusion has already been shown to be effective in persons with SCI (25, 26).

The primary aim of this scoping review is to provide an overview over the existing psychosocial digital interventions for individuals with SCI. We work out how these interventions are organized in terms of indication and application areas, theory basis, human support, and technology, and describe which characteristics of the digital interventions might have beneficial outcomes.

## 2 Methods

For this scoping review, the literature was systematically screened in accordance with the PRISMA Extension for Scoping Reviews (PRISMA-ScR) (27), except that a research protocol was not previously registered (see Supplementary Table S1).

### 2.1 Review process

All literature searches were performed with regard to previously defined search criteria (see below) by two independent reviewers (AA and KO). Search results were compared and in case of different decisions regarding an article, inclusion or exclusion was discussed to come to an agreement. Duplicate articles, articles published not in English or German, and articles other than original studies were excluded. No articles that were published before 2012 were found that met the inclusion and exclusion criteria. In order to reflect the current state of research, it was decided to exclude articles published before 2012. Titles of all remaining articles were screened for eligibility, and abstracts were screened if the title did not suffice for a decision.

### 2.2 Search and eligibility criteria

**Figure 1:**
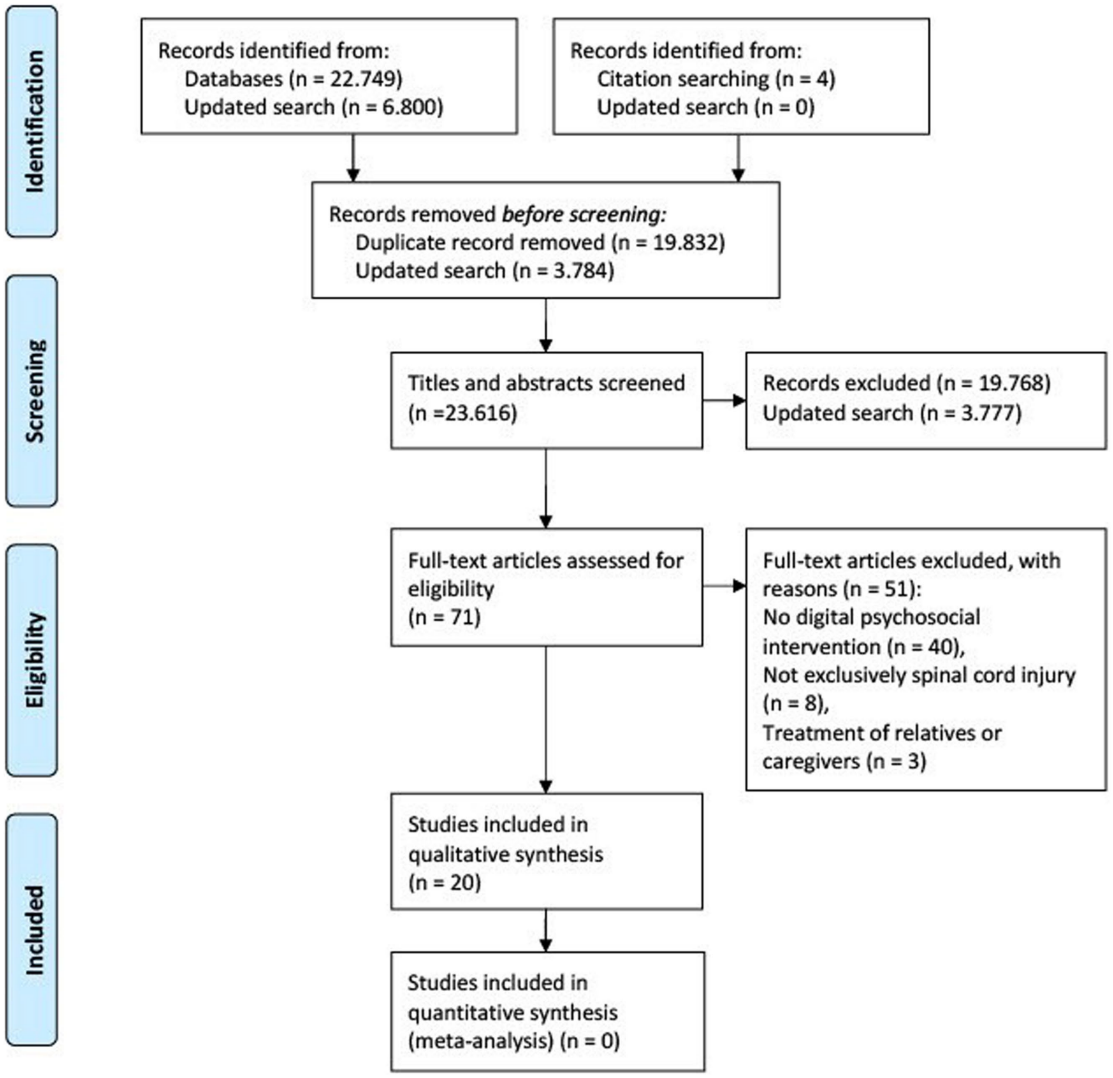
PRISMA flow chart illustrating the original selection process for the relevant studies included in this literature review (27).

The initial electronic literature search was conducted between September 2021 and January 2022 and was updated in May 2023 before submission. The databases PubMed, PsycInfo and PSYNDEX were screened for the following search terms: ((spinal cord injur*) OR (spinal injur*) OR (paraplegi*) OR (tetraplegi*) OR (quadriplegi*) OR (sci)) AND ((internet*) OR (web) OR (virtual*) OR (apps) OR (application*) OR (video*) OR (online*) OR (ehealth) OR (etherapy) OR (mhealth) OR (mtherapy) OR (mobile health) OR (phone*)) AND ((pain) OR (depression*) OR (anxiety) OR (self-management) OR (self-efficacy) OR (quality of life) OR (satisfaction) OR (stress*)). These search terms revealed 22,749 journal articles. Further four articles were identified through screening of reference lists. Sixty-four relevant articles were identified after duplicates were removed and the titles and abstracts of each article were reviewed. After screening of the full texts and the reference lists of relevant articles, a total of 18 articles were included in this literature review. The update in May 2023 revealed two more relevant articles. The studies had to meet the following eligibility criteria: German or English language, exclusively SCI, psychosocial treatment of people with SCI and not their relatives or caregivers. The original study selection and screening process is presented in the PRISMA 2020 flowchart (Figure 1).

### 2.3 Data synthesis and extraction

Studies found were assessed according to Paganini et al.’s (15) characteristics and presented in the results section. Additionally, the following data of eligible articles were extracted and presented in Supplementary Tables S2–S5: first author and publication year of article, name of psychosocial digital intervention, study design (pretest-posttest design, randomized controlled trial), country, form of guidance, dropout rate, psychosocial outcome measures, and effects on outcomes.

### 2.4 Risk of bias assessment

**Figure 2:**
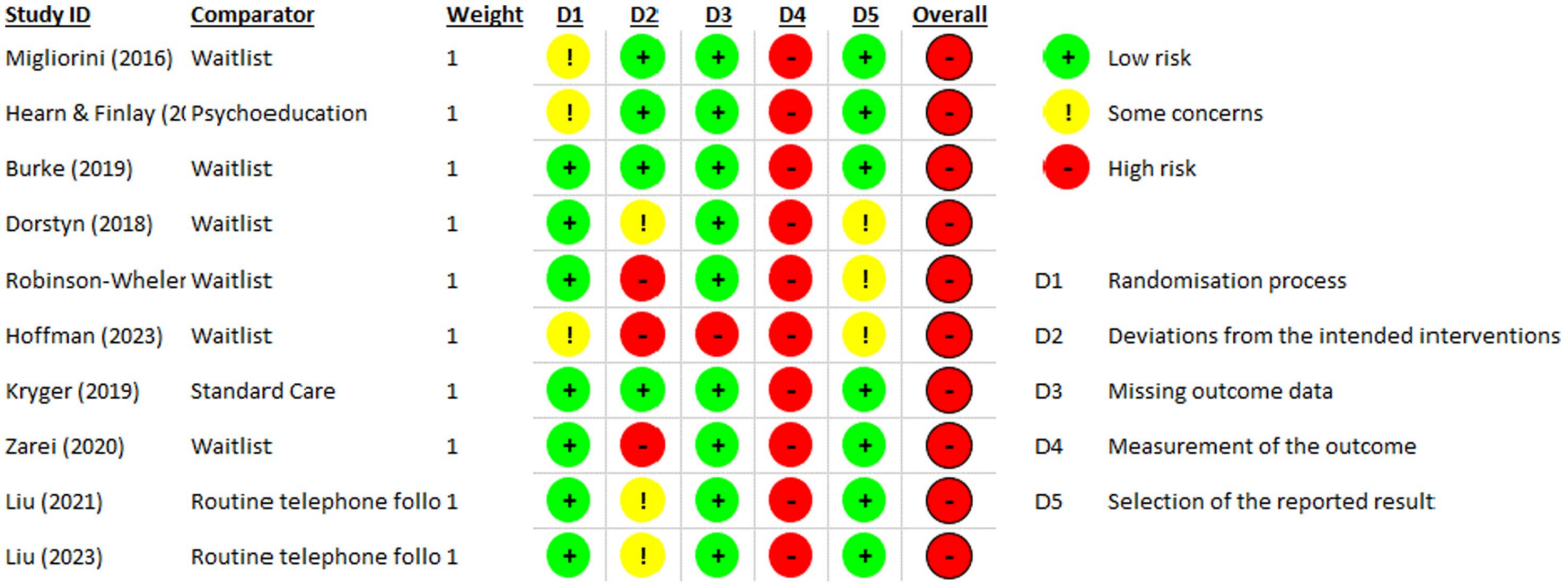
Risk of bias of included randomized-controlled studies.

The risk of bias of included randomized-controlled studies was assessed by using the Cochrane risk-of-bias tool for randomized trials (RoB 2) (28) with regard to a fixed set of domains of bias: Randomization process, Deviations from the intended interventions, Missing outcome data, Measurement of the outcome, and Selection of the reported result. These domains were evaluated in terms of low risk of bias (+), some concerns (!) and high risk of bias (−). The results of the risk of bias assessments are reported in Figure 2.

## 3 Results

The search resulted in twenty articles, which are described in more detail in Supplementary Tables S2–S5. Due to the heterogeneity of the identified digital psychosocial interventions for individuals with SCI, they are assessed using the four categories suggested by Paganini et al. (15): *indication & application areas, theory basis, structure & human support and technology.* The studies need to be considered under these parameters to determine what can be recommended for further research.

### 3.1 Indication and application areas

All digital psychosocial interventions were used exclusively as aftercare measures or could not be clearly categorised as preventive, acute or aftercare measures. For example, interventions to promote self-esteem (29) or self-management (30–36) are applicable at every stage of treatment from prevention to relapse prevention. Nineteen interventions were carried out as stand-alone interventions without further therapeutic support, while one intervention was supported by two weekly psychological counselling sessions (37).

### 3.2 Theory basis

The studies show clear differences in their theoretical basis. While some interventions were not based on any theory, CBT served as the predominant theoretical basis in the digital psychosocial interventions for people with SCI (31, 38–42). The basics of mindfulness were applied in four online programs (41–44). Two interventions were grounded in social learning theory (29, 36), one intervention in dialectical behavioral therapy (DBT) (43) and one intervention in problem-solving therapy (42). Furthermore, some studies applied components of positive psychology (41, 42), and feminist psychology (29).

### 3.3 Structure and human support

In order to assess the quality of the digital interventions for individuals with SCI, it is important to determine whether the digital interventions have been developed or adapted to the clinical picture of SCI. In 17 studies, the respective digital interventions were adapted to the needs and barriers of individuals with SCI. This tailoring involved for example adding SCI-specific examples, case stories, information about SCI and feasible exercises. However, two authors discussed the lack of adaptation as limiting factor (42, 45) and one article did not provide any information on being adapted or not (32).

The number of digital intervention modules ranged from 2 to 72, with most interventions containing five to seven modules. While some applications had a set time frame, e. g. one module per week, others were self-directed by the participants. A distinction must also be made if the modules included in the interventions are mandatory or optional. There were also interventions with fixed meetings or appointments.

The guidance, which was provided for example by a clinical psychologist or a peer, varied greatly between the online programs. In two studies, participants had weekly contact with a clinical psychologist via phone or e-mail (39, 40). In one intervention, weekly contact with a researcher was offered by telephone (42), and in another intervention there was weekly contact during inpatient treatment and 1–2 times per month after discharge (35). One intervention was assisted by a psychotherapist (43) and another one was facilitated by a psychologist and a peer (29). Three interventions were solely accompanied by a peer, for example via video chat (30, 31, 36). Further applications were supported by trained nursing staff (33, 34), a physiotherapist (37) or a music therapist (46). The intervention of another study offered a peer forum and telephone or e-mail contact by a multidisciplinary team specialized in SCI (38). While support by phone or e-mail was optional in two interventions (32, 41), four studies did not mention any personal guidance or support and have only offered educational content or mindfulness exercises digitally (44, 45, 47, 48).

### 3.4 Technology

Twelve internet and mobile-based interventions, five smartphone apps and three virtual reality applications were revealed by the literature search. The internet- and mobile-based interventions can be grouped into *internet- and mobile-based interventions for mental health* and *internet- and mobile-based interventions for other life aspects,* for example concerning work or self-esteem. The studies were very heterogeneous in terms of their psychosocial outcome measures and their results. Supplementary Tables S2–S5 present the outcomes of the digital psychosocial interventions, categorized according to the technological implementation of the intervention.

The study designs involve ten randomized controlled trials and ten non-randomized controlled trials. The non-RCTs can be grouped into four single-group pre-post studies, three mixed-methods studies and two case studies. Another study used a two-phase iterative design. The study sample sizes ranged from 1 to 184. Five studies included individuals with traumatic SCI cause only and six studies did not mention the cause of SCI.

### 3.5 Risk of bias

Results of the Risk of bias assessment for randomized trials (see 2.4) are reported in Figure 2. All ten RCT report high overall risk of bias due to the digital intervention approach and the study designs. Digital interventions generally have high drop-out rates (49, 50), which can be due to various factors such as improvement, age and health literacy (50), and result in a high risk of bias since outcome data is missing. Furthermore, all studies report a high risk of bias in the domain “measurement of the outcome”, as blinding was not possible due to the study designs and the outcomes were self-reported. Therefore, when collecting data, participants may have been biased.

## 4 Discussion

The aim of this review is to highlight the care landscape of digital psychosocial interventions for people with SCI. Twelve Internet and mobile-based interventions, five apps and three virtual reality applications have already been developed for these individuals and evaluated in at least 20 randomized and non-randomized clinical trials. Despite a lack of strong evidence based studies, online applications have the potential to enrich the mental health and well-being of people with SCI.

Almost all digital interventions were offered as stand-alone programs and only one study was conducted with accompanying psychological counselling. The programs were mainly used as aftercare measures, but some interventions could also be used at any stage of treatment, from prevention to relapse prevention. Whilst some of the digital interventions were not based on any theory, CBT mostly served as the theoretical basis for the online interventions, alongside other foundations such as mindfulness, social learning theory or DBT. Depending on the intervention, between 2 and 72 modules were offered, with 5–7 in most cases. These were either optional or mandatory and were conducted within a set time frame or self-directed. Seventeen of the total of 20 interventions were adapted to people with SCI, for example by adding case stories. In the studies, there was either no human support, or guidance from a psychologist, peer or other professional groups (e. g. trained nurses). The guidance was carried out in different time frames, either by phone, e-mail, video chat, forums or live. While support was a required element in some studies, it was optional in others.

Various effects in individuals with SCI were reported by the application of different digital interventions. The comparability of the studies and thus of the online programs is limited by the different intervention approaches and different outcome parameters.

While psychosocial virtual reality applications for people with SCI show promising results, it is too early to make recommendations, as these need to be further validated in larger study samples. Regarding IMIs and apps, the results can be attributed to various factors. Interventions based on a theory such as CBT in particular have achieved their goals (31, 38–42). In addition, professional human support, e.g., by phone or mail, appears to be a favourable factor: participants seem to benefit more from professional support, for example from a clinical psychologist (32–35, 37–43), than from peer support (29–31, 36, 46) or no support (44, 45, 47, 48). Adaptation of the digital interventions to people with SCI can also be recommended as lack of adaption was a main point of criticism in two interventions (42, 45).

CBT as an evidence-based theoretical foundation was well suited as an online-based intervention due to its directiveness and standardisation. A meta-analysis by (51) confirmed that it had a significant positive impact on short-term psychological outcomes after SCI. Significant outcomes were found more frequently in these studies than in programmes without a clear structure (51).

Particularly regarding IMIs, the studies showed that that there was a slight advantage for those who had regular intervals of professional therapist contact in addition to theoretical grounding (39, 40). The support of a psychologist can possibly be seen as the equivalent of the therapeutic relationship of classical psychotherapy and should be considered in the development of further digital interventions. This finding is in line with the meta-analysis of Mehta et al. (52), where guided support is associated with a better understanding and implementation. Another facilitating factor is the “supportive accountability,” as reported by Domhardt and Baumeister (53).

The duration of the interventions was another variable that influenced the outcomes of the interventions. Those studies that had modules developed at regular intervals over a longer period of time (> 8 weeks) (39–41, 44) recorded more varied and sometimes greater effects than interventions that let their users control the programme themselves (30–32, 35, 47, 48). However, it is yet unclear how many modules with which content are needed to achieve improvements at the psychosocial level, and in addition to lower dropout rates which differed greatly between the digital interventions (0%–50%).

The wide range of dropouts is in line with the meta-analysis by Meyerowitz-Katz et al. (50), according to which the dropout rate of *mhealth* apps in the general population is widely spread, averaging 43%. In the studies of this review, dropout was caused, for example, by lack of adaption to the needs of people with SCI (32, 42, 45), inadequate internet access (35), or inability to use the app independently (35). However, individuals with different levels of SCI were able to use the digital interventions, with some requiring assistive devices such as hand pens, mouth pens or additional human support (35).

Adherence is a crucial aspect of digital interventions, as, according to Donkin et al. (54), there is a correlation between module completions and the outcomes of psychological interventions. This assertion is in line with Domhardt and Baumeister (53), which postulate that patient engagement is central to creating change, especially in the absence of real contact with a mental health professional. This highlights the relevance of further studies to promote engagement to internet-based therapies. One way to increase the use of online interventions is through so-called prompts or reminders (55).

Three applications that evaluated virtual reality in the context of therapeutic treatment for people with SCI were included in this review (37, 43, 46). In addition to the preliminary effects on psychological outcomes, immersive VR technology using VR glasses allows individuals, who spend a lot of time in bed and in hospital during the first months of rehabilitation, to escape from everyday life and to establish and maintain social contacts, especially for people in rural areas or in times of the Covid-19 pandemic (46). Another positive aspect of immersive VR applications is that users are distracted from pain and other concerns (25, 26). Maresca et al. (37) combined a treatment of face-to-face psychological treatment, cognitive training, and physiotherapy in conjunction with cognitive and motor VR modules. This approach is also interesting as it highlights that conventional therapy can be complemented by the integration of virtual reality and can equally lead to significant improvements in psychosocial aspects such as depressive disorders. However, this conclusion needs to be considered cautiously, as the results from a single subject are not conclusive.

### 4.1 Limitations

The aim of this scoping review was to provide an overview of the landscape of provision of digital psychosocial interventions for people with SCI, which may not have included some online interventions on other databases or in other languages. Studies on IMIs, apps and VR interventions are rapidly emerging, which means that the effects found may no longer represent the current state of research or new applications may have already been developed. Studies that included concrete online psychosocial interventions were included in this work. However, it cannot be ruled out that other treatment approaches such as exercise can also have positive effects on the mental health of people with SCI.

Another limitation is that due to the heterogeneity of study designs and intervention approaches, meta-analytic evaluations are not conducted. In their meta-analysis, Blackport et al. (56) identified five studies on internet-based psychosocial interventions for people with SCI. These also report significant effects in the areas of depression, anxiety and pain.

The generalisability of the psychological and social effects found on the subjects is also limited by the limitations of the individual studies. Many studies had samples that were too small and homogeneous or did not compare the results with a control group. The subjects recruited were interested in digital treatment and thus may not have formed a representative SCI population. The follow-up periods of three months were not sufficient for the majority of the studies to be able to make statements about long-term changes. In addition, different outcomes were intended, and different measurement instruments were used.

### 4.2 Implications for further research

There is still uncertainty about both the impact of different qualifications of the professional as well as the optimal modality of contact (e. g. e-mail or telephone). Also, the duration required for significant effects on psychosocial parameters is yet unclear. Furthermore, it is important to further investigate which specific contents in which module length were decisive for success. The adequate degree of structuredness or flexibility is also an important variable that still needs to be examined.

The concept of “tailoring” internet-based interventions is exciting and worth further development, especially for individuals with SCI, as they could individually benefit from adapted module content due to different levels of SCI and the resulting needs and limitations. The studies presented in this review have not yet analyzed the individual needs of the persons with SCI who took part in the respective digital interventions.

This review highlights the diverse possibilities of digital interventions for people with SCI, but indicates that further large-scale research, especially randomized controlled trials, is essential to determine which features are crucial for their use in this group of people with special needs, the long-term effects and to generalise the effects found to be translated into clinical practice.

## 5 Conclusion

The process of coping with SCI can benefit from psychological support. In addition to standard face-to-face treatment, various digital psychosocial applications (internet- and mobile-based interventions, smartphone apps, virtual reality interventions) have already been developed specifically for people with SCI. Factors that positively influence the outcomes include evidence-based theoretical foundation, human support, structure and regular module frequency. Individuals with SCI are nevertheless underserved in the field of psychosocial digital interventions due to a lack of high-quality studies.

## Data availability statement

The original contributions presented in the study are included in the article/Supplementary material, further inquiries can be directed to the corresponding author.

## Author contributions

AA: Data curation, Formal analysis, Investigation, Methodology, Visualization, Writing – original draft. KO: Conceptualization, Investigation, Methodology, Writing – review & editing. KW: Formal analysis, Methodology, Writing – review & editing. KH: Conceptualization, Methodology, Project administration, Supervision, Writing – review & editing.
