# Supplementary material for: Digital psychosocial interventions for individuals with spinal cord injury: a scoping review
Source: Front Psychiatry. 2024 Jan 22;15:1289138. doi: 10.3389/fpsyt.2024.1289138 (PMC10838963; doi:10.3389/fpsyt.2024.1289138)
Supplement: Supplementary file 1 [file Table_1.docx]

Supplementary Material

Digital psychosocial interventions for individuals with spinal cord injury: a scoping review

**Alice Armstrong, Katja Oetinger*, Katja Weimer, Klaus Hönig**

*** Correspondence:**

Katja Oetinger (Reichert)

([katja.reichert@uni-ulm.de](mailto:katja.reichert@uni-ulm.de))

**Index:**

Table 1: PRISMA checklist

Table 2: Summary of internet- and mobile-based interventions for mental health

Table 3: Summary of psychosocial internet- and mobile-based interventions for other life aspects than mental health

Table 4: Summary of smartphone apps

Table 5: Summary of virtual reality applications

**Table 1: PRISMA checklist, according to (27).**

| **SECTION** | **ITEM** | **PRISMA-ScR CHECKLIST ITEM** | **REPORTED ON PAGE #** |
| --- | --- | --- | --- |
| **TITLE** | | | |
| Title | 1 | Identify the report as a scoping review. | 1 |
| **ABSTRACT** | | | |
| Structured summary | 2 | Provide a structured summary that includes (as applicable): background, objectives, eligibility criteria, sources of evidence, charting methods, results, and conclusions that relate to the review questions and objectives. | 2 |
| **INTRODUCTION** | | | |
| Rationale | 3 | Describe the rationale for the review in the context of what is already known. Explain why the review questions/objectives lend themselves to a scoping review approach. | 3-4 |
| Objectives | 4 | Provide an explicit statement of the questions and objectives being addressed with reference to their key elements (e.g., population or participants, concepts, and context) or other relevant key elements used to conceptualize the review questions and/or objectives. | 4 |
| **METHODS** | | | |
| Protocol and registration | 5 | Indicate whether a review protocol exists; state if and where it can be accessed (e.g., a Web address); and if available, provide registration information, including the registration number. | 4 |
| Eligibility criteria | 6 | Specify characteristics of the sources of evidence used as eligibility criteria (e.g., years considered, language, and publication status), and provide a rationale. | 4 |
| Information sources* | 7 | Describe all information sources in the search (e.g., databases with dates of coverage and contact with authors to identify additional sources), as well as the date the most recent search was executed. | 4-5 |
| Search | 8 | Present the full electronic search strategy for at least 1 database, including any limits used, such that it could be repeated. | 5 |
| Selection of sources of evidence† | 9 | State the process for selecting sources of evidence (i.e., screening and eligibility) included in the scoping review. | 16 |
| Data charting process‡ | 10 | Describe the methods of charting data from the included sources of evidence (e.g., calibrated forms or forms that have been tested by the team before their use, and whether data charting was done independently or in duplicate) and any processes for obtaining and confirming data from investigators. | / |
| Data items | 11 | List and define all variables for which data were sought and any assumptions and simplifications made. | 5 |
| Critical appraisal of individual sources of evidence | 12 | If done, provide a rationale for conducting a critical appraisal of included sources of evidence; describe the methods used and how this information was used in any data synthesis (if appropriate). | 5 |
| Synthesis of results | 13 | Describe the methods of handling and summarizing the data that were charted. | 5 |
| **RESULTS** | | | |
| Selection of sources of evidence | 14 | Give numbers of sources of evidence screened, assessed for eligibility, and included in the review, with reasons for exclusions at each stage, ideally using a flow diagram. | 16 |
| Characteristics of sources of evidence | 15 | For each source of evidence, present characteristics for which data were charted and provide the citations. | 5-7 |
| Critical appraisal within sources of evidence | 16 | If done, present data on critical appraisal of included sources of evidence (see item 12). | 7 |
| Results of individual sources of evidence | 17 | For each included source of evidence, present the relevant data that were charted that relate to the review questions and objectives. | 5-7 |
| Synthesis of results | 18 | Summarize and/or present the charting results as they relate to the review questions and objectives. | 7 |
| **DISCUSSION** | | | |
| Summary of evidence | 19 | Summarize the main results (including an overview of concepts, themes, and types of evidence available), link to the review questions and objectives, and consider the relevance to key groups. | 7 |
| Limitations | 20 | Discuss the limitations of the scoping review process. | 9 |
| Conclusions | 21 | Provide a general interpretation of the results with respect to the review questions and objectives, as well as potential implications and/or next steps. | 9-10 |
| **FUNDING** | | | |
| Funding | 22 | Describe sources of funding for the included sources of evidence, as well as sources of funding for the scoping review. Describe the role of the funders of the scoping review. | 10 |

**Table 2: Summary of internet- and mobile-based interventions for mental health**

| **First author** | **Psychosocial digital intervention** | **Study design (*N*)** | **Country** | **Guidance** | **Dropout** | **Psychosocial outcome measures** | **Outcomes** |
| --- | --- | --- | --- | --- | --- | --- | --- |
| Stuntzner (2015) | Forgiveness is a Choice, Coping Effectively Training | Pretest-posttest design (*N* = 16) | USA | Voluntary contact | 31% | BDI-II, STAI, STAXI-II | Forgiveness group: Statistically significant decrease in depression (*p* < .05), trait anxiety (*p* < .01) and trait anger (*p* < .05). There were no significant changes in state anxiety and state anger.  Coping group: Statistically significant decrease in depression (*p* < .01) and state anxiety (*p* < .05) but not in trait anxiety, state anger or trait anger.  Both interventions were comparable in changes in depression, state and trait anxiety and state and trait anger (*p* > .05). |
| Migliorini (2016) | ePACT | Randomized-controlled trial (*N*= 59) | Australia | Voluntary contact | 19% | DASS21, PWI-A, SCL EWL | Intervention group: Univariate within group analysis showed significant improvements in depression (*η^2^* = 0.37), anxiety (*η^2^* = 0.43), stress (*η^2^* = 0.28) and satisfaction with life (*η^2^* = 0.24) at post-treatment. At follow-up, 6 months post intervention, (N =12) depression, anxiety and stress decreased substantially by the post-intervention time point. Satisfaction with life scores did not change by follow-up.  Control group: The analysis showed significant improvements in depression only (*η^2^* = 0.25). The control group did not take part at follow-up. |
| Verwer (2016) | Psyfit | Pretest-posttest design (*N* = 14) | The Nether-lands | Contact every two weeks | 50% | MHI-5, CES-D, WEMWBS | The completers (N =7) reported a significant improvement of the MHI scores (within group Pearson´s *r* = .53) and a non-significant change in the WEMWBS scores at post-treatment. The changes were not maintained at 3-month follow-up. All scores decreased significantly between post-treatment and follow-up, and no significant change was seen between T1 and T3. |
| Dear (2018) | The Pain Course | Pretest-posttest design (*N* = 68) | Australia | Weekly contact | 24% | Primary: PDI, PHQ-9, GAD-7  Secondary: WBPQ; Tertiary: PSEQ, PCS, SWLS | Primary: The analyses revealed a significant time effect for pain-related disability (within group Cohen´s *d* = 0.53, CI = 0.53-0.88), depression (*d* = 0.44, CI = 0.45-0.79) and anxiety (*d* = 0.41, CI = 0.42-0.76) at post-treatment. At 3-month follow-up, the effects improved further significantly in pain-related disability (*d* = 0.71, CI = 0.68-1.06), depression (*d* = 0.76, CI = 0.82-1.12) and anxiety (*d* = 0.63, CI = 0.67-0.98).  Secondary: A significant overall time effect for average pain intensity was found at post-treatment (*d* = 0.46, CI = 0.44-0.81), but no change was found from post-treatment to follow-up.  Tertiary: Significant overall time effects were found for pain self-efficacy (*d* = 0.11, CI = –0.24-0.11), pain catastrophising (*d* = 0.54, CI = 0.58-0.89) and satisfaction with life (*d* = 0.31, CI = –0.04-0.30) at post-treatment. Satisfaction with life further improved significantly from post-treatment to follow-up (*d* = 0.55, CI = 0.19-0.52), while pain catastrophising did not change over this time. Pain self-efficacy did not improve between baseline and post-treatment but did improve from post-treatment to 3-month follow-up (*d* = 0.36, CI = 0.01-0.35). |
| Hearn & Finlay (2018) | The Mindfulness Course | Randomized-controlled trial (*N =* 67) | United Kingdom | No contact | 36% | HADS, WHOQOL-BREF, FFMQ, NRS, PCS | Mindfulness training led to significant reductions in depression (*η^2^_p_* = 0.18), anxiety (*η^2^_p_* = 0.14), pain unpleasantness (*η^2^_p_* = 0.14) and pain catastrophizing (*η^2^_p_* = 0.11) at post-intervention. Likewise, there were significant improvements in the total FFMQ score (*η^2^_p_* = 0.28) and nearly all facets of mindfulness (*η^2^_p_* = 0.08-0.22) at post-intervention, when compared with psychoeducation.  No significant group differences were found post-intervention for any aspect of quality of life, pain intensity, and mindfulness facets of observing and non-judging.  At 3-month follow-up, reductions in depression (*η^2^_p_* = 0.22), anxiety (*η^2^_p_* = 0.22) and pain catastrophizing (*η^2^_p_* = 0.24) persisted. |
| Burke (2019) | SPIRE | Randomized-controlled trial (*N*= 69) | Ireland | Voluntary contact, Peer-Forum, Live-Webinar | 26% | Primary: WHOQOL-BREF  Secondary: ISCIQOLBDS, ISCIPBDS, DN4, CPAQ-8, BPI interference sub-scale, HADS, PSQI; PGIC | Primary: There was no significant difference over time between the intervention and control groups in quality of life.  Secondary: The intervention demonstrated a significant group x time interaction for NRS of overall pain (T1-T2: *d* = 0.70, CI = 0.16-1.24; T1-T3: *d* = 0.38, CI = 0.18-0.94), worst pain (T1-T2: *d* = 0.84, CI = 0.30-1.39; T1-T3: *d* = 0.55, CI = -0.01-1.11), and for Brief Pain Inventory (interference) (T1-T2: *d* = 0.53, CI = -0.00-1.07; T1-T3: *d* = -0.28, CI = -0.84-0.27). No significant group x time interaction was found for mood, sleep and pain acceptance. Post intervention, 34% of participants reported being much or very much improved. At the 3-month follow-up, 48% of participants reported being much or very much improved. |
| Mehta (2020) | The Chronic Conditions Course | Pretest-posttest design (*N* = 20) | Canada | Weekly contact | 10% | Primary: PHQ-9, GAD-7  Seconary: ISCIBPDS | Primary: Significant improvements in depression (*χ^2^* = 18.27, *p* < .01) and anxiety (*χ^2^* = 12.72, *p* < .01) were found in within subjects analysis from baseline to post-treatment and from baseline to follow-up. Further significant reductions were seen in depression scores from post-treatment to follow-up (*p* < .05), while no significant difference in anxiety scores was seen from post-treatment to follow-up.  Secondary: Overall significant time effects were found on SCIQoL subscales of Grief (Wald’s *χ2* = 19.38, *p* < .001), Self-Esteem (Wald’s *χ2* = 6.62, *p* = .04), Resilience (Wald’s *χ2* = 14.11, *p* < .002), Positive Affect (Wald’s *χ2* = 15.92, *p* < .001), and Ability to participate (Wald’s *χ2* = 15.68, *p* < .001), but not in Independence subscale. |

***Note.*** Abbreviations: *BDI-II* Beck Depression Inventory-II*, STAI* State-Trait Anxiety Inventory*, STAXI-II* State-Trait Anger Expression Inventory-II*, DASS21* Depression, Anxiety and Stress Scale Short Version*, PWI-A* Personal Well-being Index-Adult*, SCL EWL* Spinal Cord Lesion Emotional Well-being Questionnaire*, MHI-5* Mental Health Index-5*, CES-D* Center of Epidemiological Studies Developmental Scale*, WEMWBS* Warwick-Edinburgh Mental Well-Being Scale*, PDI* Pain Disability Index*, PHQ-9* Patient Health Questionnaire-9*, GAD-7* General Anxiety Disorder-7, *WBPQ* Wisconsin Brief Pain Questionnaire*, PSEQ* Pain Self-Efficacy Questionnaire*, PCS* Pain Catastrophising Scale*, SWLS* Satisfaction With Life Scale*, HADS* Hospital Anxiety and Depression Scale*, WHOQOL-BREF* World Health Organization Quality of Life Brief Instrument*, FFMQ* Five Facet Mindfulness Questionnaire*, NRS* Numerical Rating Scale*, PCS* Pain Catastrophizing Scale*, ISCIQOLBDS* International Spinal Cord Injury Quality of Life Basic Data Set*, ISCIPBDS* International Spinal Cord Injury Pain Basic Data Set*, DN4* Douleur Neuropathique en 4 Questions Interview*, CPAQ-8* Chronic Pain Acceptance Questionnaire-8*, BPI* Brief Pain Inventory*, PSQI* Pittsburgh Sleep Quality Index*, PGIC* Participants´ Global Impression of Change.

**Table 3: Summary of psychosocial internet- and mobile-based interventions for other life aspects than mental health**

| **First author** | **Psychosocial digital intervention** | **Study design (*N*)** | **Country** | **Guidance** | **Dropout** | **Psychosocial outcome measures** | **Outcomes** |
| --- | --- | --- | --- | --- | --- | --- | --- |
| Dorstyn (2018) | Work & SCI | Randomized-controlled trial (*N*= 48) | Australia | No contact | 36% | Primary: JSES  Secondary: LOT-R, PHQ-9 | No significant main or interaction effects for the outcomes were found.  A small improvement in optimism (*g* = 0.24, CI = 0.04-0.44) in comparison to the control group (*g* = −0.04, CI = −0.29-0.21) was found four weeks after enrolment.  81% evaluated work alternatives, 56% researched job opportunities and 44% discussed their options with friends or family. |
| Newman (2018) | PHOENIX | Cross-sectional Pilot study (*N* = 10) | USA | Peer-Support | / | / | The aim of the study was to investigate the feasibility and acceptance of the intervention. Despite technical difficulties encountered, both aspects were fulfilled. |
| Allin (2020) | SCI & U | Mixed methods Pilot study (*N* = 11) | Canada | Peer-Support | 9% | UW-SES, SCI-QOL-R, PHQ-8 | Evidence points to an improvement in depressive symptoms (PHQ-8: within group Hedge´s *g* = 0.30, CI = –0.11-0.71), resilience (SCI-QOL-R: *g* = –0.36, CI = –0.6- –0.10) and self-efficacy (UW-SES: *g* = –0.52, CI = –1.08-0.03) at post-test, but no significant effects were found. |
| Robinson-Whelen (2020) | SEE-SCI | Randomized-controlled trial (*N*= 21) | USA | Weekly contact and Peer-support | 8.7% | HPLP-II 3 subscales, MOS-SS subscale, GSES, RSES, CESD-10, PHQ-9 | The intervention group had significantly greater improvements in Interpersonal Relations and Spiritual Growth/Self-Actualization than the control group (*p* < .05). No effect on the Stress Management subscale or other outcome was seen.  Within the intervention group, there were significant improvements in interpersonal relationships (*d* = 0.72) and spiritual growth (*d* = 0.92). Furthermore, significant reductions in depressive symptoms (CESD-10: *d* = 0.76; PHQ-9: *d* = 0.79) were reported. No statistically significant effects were found in social support, generalized self-efficacy or self-esteem. |
| Hoffman (2023) | SCI Thrive | Randomized-controlled trial (*N*= 184) | USA | Peer- and participant-support | 9% | Primary: PQOL  Secondary: SMCD, LSA, CHART | Primary: No statistically significant changes were seen in PQOL.  Secondary: Compared to the waitlist group, the treatment group had significantly higher scores on CHART occupational subscale (*p* < .05), but no other differences were found at the end of 6 weeks. Analysis of all participants who completed SCI Thrive showed significant increase in self-efficacy between baseline and 6 weeks (*p* < .001) which was maintained at 3 months follow-up (*p* < .001). |

***Note.*** Abbreviations: *JSES* Job Procurement Self-Efficacy Scale*, LOT-R* Life Orientation Test-Revised*, PHQ* Patient Health Questionnaire*, UW-SES* University of Washington Self Efficacy Scale*, SCI-QOL-R* Spinal Cord Injury Quality of Life Resilience Scale*, HPLP-II* Health Promoting Lifestyle Profile-II*, MOS-SS* Medical Outcomes Study Social Support Survey*, GSES* Generalized Self-Efficacy Scale*, RSES* Rosenberg Self-Esteem Scale*, CESD-10* Centre for Epidemiologic Studies Depression Scale-10, *PQOL* Perceived Quality of Life Scale, *SMCD* Self-Efficacy for Managing Chronic Disease 6-item Scale, *LSA* Life Space Assessment, *CHART* Craig Handicap Assessment and Reporting Technique.**Table 4: Summary of smartphone apps**

| **First author** | **Psychosocial digital intervention** | **Study design (N)** | **Country** | **Guidance** | **Dropout** | **Psychosocial outcome measures** | **Outcomes** |
| --- | --- | --- | --- | --- | --- | --- | --- |
| Kryger (2019) | iMHere | Randomized-controlled trial (*N*= 38) | USA | Voluntary contact | 13% | COPM, ASIS, BDI-II, PACIL, WHOQOL-BREF, CHART-SF physical independence domain | The app did not lead to significant psychosocial changes, but there was evidence of improvement in depressive symptoms. |
| MacGillivray (2020) | SCI-Storylines | Feasibility study (*N* = 20) | Canada | Regularly contact (every 1-2 weeks) | 30% | HADS | Over admission, discharge and 3-month post discharge, HADS scores have remained in the normal range, with an average score below 7. |
| Zarei (2020) | SAMAR | Randomized-controlled trial (*N*= 70) | Iran | No contact | 0% | SAQ, LSSS, SMAS, EMSS | The intervention group reported significant improvements (*p* < .01) in sexual adjustment (mean difference = 4.2, CI = 3.6-4.8, *η^2^_p_* = 0.18), sexual satisfaction (mean difference = 9.6, CI = 8.0-11.2, *η^2^_p_* = 0.44) and martial satisfaction (mean difference = 4.1, CI = 3.3-4.9, *η^2^_p_* = 0.08). There was no significant increase in martial adjustment´s between-group mean difference after four weeks. |
| Liu (2021) | Together | Randomized-controlled trial (*N*= 98) | China | Contact during sessions | < 1% | MSES, SF-36 | The intervention led to improvements in self-efficacy over time (T0 = 67.80, T1 = 71.90, and T2 = 76.29) and was higher than the control group (T2 = 64.49) at 24 weeks following discharge (*p* < .01). The intervention group reported higher scores in quality of life (T2 = 65.36) than the control group (T2 = 85.77), but only time effects were significant (*p* < .01). |
| Liu (2023) | Together | Randomized-controlled trial (*N*= 98) | China | Contact during sessions | < 1% | BDI-II | The intervention group had lower levels of depression at T2 (*p* < .01). Small to moderate effect sizes on depression favouring the intervention were demonstrated at T1 (*d* = -0.18, CI = -0.57-0.22) and T2 (*d* = -0.54, CI = -0.94- -0.13). |

***Note.*** Abbreviations: *COPM* Canadian Occupational Performance Measure*, ASIS* Adolescent Self-Management and Independence Scale*, BDI-II* Beck Depression Inventory-II*, PACIL* Patient Assessment of Chronic Illness Care*, WHOQOL-BREF* World Health Organization Quality of Life Brief Instrument*, CHART-SF* Craig Handicap Assessment and Reporting Technique Short Form*, HADS* Hospital Anxiety and Depression Scale*, SAQ* Sexual Adjustment Questionnaire*, LSSS* Larsons´s Sexual-Satisfaction Scale*, SMAS* Spinner´s Marital Adjustment Scale*, EMSS* ENRICH Marital Satisfaction Scale*, MSES* Moorong Self-Efficacy Scale*, SF-36* Short-Form Health Survey.

**Table 5: Summary of virtual reality applications**

| **First author** | **Psychosocial digital intervention** | **Study design (N)** | **Country** | **Guidance** | **Dropout** | **Psychosocial outcome measures** | **Outcomes** |
| --- | --- | --- | --- | --- | --- | --- | --- |
| Flores (2018) | DBT Mindfulness | Case study (*N*= 2) | USA | Assistance during session | 50% | BDI-7, STAI-Y, GRS, NRS | After the intervention, all patients reported feeling less depressed, less anxious and less emotionally upset.  Patient 1 had reductions in negative emotions after each session.  Patient 2 reported a decrease in short term ASD/PTSD symptoms but had mixed outcomes on some of the measures of negative emotions. |
| Maresca (2018) | VRRS Training (Combined rehabilitative training) | Case study (*N*= 1) | Italy | Assistance during session | 0% | HRS-D, HRS-A | The intervention led to a reduction in depression and anxiety symptoms. |
| Tamplin (2019) | Group Singing | Feasibility study (*N* = 12) | Australia | Assistance during session | / | PIADS | A mildly positive impact of the VR equipment on psychosocial outcomes were indicated. |

***Note.*** Abbreviations**:** *BDI-7* Beck Depression Inventory-Fast Screen*, STAI-Y* State-Trait Anxiety Inventory*, GRS* Graphic Rating Scale, *NRS* Numeric Rating Scale*, ASD* Acute Stress Disorder, *PTSD* Post-Traumatic Stress Disorder, *HRS-D* Hamilton Rating Scale for Depression*, HRS-A* Hamilton Rating Scale for Anxiety*, PIADS* Psychosocial Impact of Assistive Devices Scale.
